# Treatment of uncomplicated *Plasmodium vivax* with chloroquine plus radical cure with primaquine without G6PDd testing is safe in Arba Minch, Ethiopia: assessment of clinical and parasitological response

**DOI:** 10.1186/s12936-023-04562-x

**Published:** 2023-04-25

**Authors:** Daniel Abebe Mekonnen, Girma Shumie Abadura, Sinknesh Wolde Behaksra, Hiwot Solomon Taffese, Gudissa Aseffa Bayissa, Mikiyas Gebremichael Bulto, Tesfaye Sisay Tessema, Fitsum G. Tadesse, Endalamaw Gadisa

**Affiliations:** 1 Malaria and Neglected Tropical Diseases Directorate, Armauer Hansen Research Institute, 1005, Addis Ababa, Ethiopia; 2 Institute of Biotechnology, Addis Ababa University, 1176, Addis Ababa, Ethiopia; 3 National Malaria Control Program/Federal Ministry of Health, Addis Ababa, Ethiopia

**Keywords:** Chloroquine, Ethiopia, *P. vivax*, Primaquine, Treatment failure

## Abstract

**Background:**

Ethiopia rolled out primaquine nationwide in 2018 for radical cure along with chloroquine for the treatment of uncomplicated *Plasmodium vivax* malaria in its bid for malaria elimination by 2030. The emergence of anti-malarial drug resistance would challenge the elimination goal. There is limited evidence on the emergence of chloroquine drug resistance. The clinical and parasitological outcomes of treatment of *P. vivax* with chloroquine plus radical cure using low dose 14 days primaquine were assessed in an endemic area of Ethiopia.

**Methods:**

A semi-directly observed 42-days follow up in-vivo therapeutic efficacy study was conducted from October 2019 to February 2020. *Plasmodium vivax* mono-species infected patients (n = 102) treated with a 14 days low dose (0.25 mg/kg body weight per day) primaquine plus chloroquine (a total dose of 25 mg base/kg for 3 days) were followed for 42 days to examine clinical and parasitological outcomes. Samples collected at recruitment and days of recurrence were examined by 18 S based nested polymerase chain reaction (nPCR) and *Pvmsp3α* nPCR-restriction fragment length polymorphism. Asexual parasitaemia and the presence of gametocytes were assessed on the scheduled days using microscopy. Clinical symptoms, haemoglobin levels, and Hillmen urine test were also assessed.

**Results:**

Of the 102 patients followed in this study, no early clinical and parasitological failure was observed. All patients had adequate clinical and parasitological responses within the 28 days of follow up. Late clinical (n = 3) and parasitological (n = 6) failures were observed only after day 28. The cumulative incidence of failure was 10.9% (95% confidence interval, 5.8–19.9%) on day 42. Among the paired recurrent samples, identical clones were detected only in two samples on day 0 and day of recurrences (day 30 and 42) using *Pvmsp3α* genotyping. No adverse effect was detected related to the low dose 14 days primaquine administrations.

**Conclusion:**

Co-administration of CQ with PQ in the study area is well tolerated and there was no recurrence of *P. vivax* before 28 days of follow up. Interpretation of CQ plus PQ efficacy should be done with caution especially when the recurrent parasitaemia occurs after day 28. Therapeutic efficacy studies with appropriate design might be informative to rule out chloroquine or primaquine drug resistance and/or metabolism in the study area.

## Background

Ethiopia achieved the Global Technical Strategy for Malaria (GTS) target; case incidence decreased by more than 40% in 2020 compared with the 2015 baseline [1]. Yet, malaria continued to be one of the public health challenges with estimated 55 million (60%) Ethiopians remaining to be at risk [2]. In 2020, Ethiopia contributed towards 1.8% (4,338,000) of the global malaria cases [1].

*Plasmodium falciparum* and *Plasmodium vivax* are co-endemic in Ethiopia, with the later contributing to ~ 40% of the total reported cases [3]. Intensified control efforts have led to major changes in malaria epidemiology [4]. *Plasmodium vivax* is a harder species to eliminate [5] partly due to its unique tendency to cause relapsing episodes related to the dormant liver stage, hypnozoites [6]. In Ethiopia, chloroquine alone was the choice of treatment for *P. vivax* [2]. Chloroquine is only a blood stage schizonticide and does not have effect on the hypnozoite stages of *P. vivax* [7]. The only World Health Organization (WHO) approved drug of choice for the treatment of the dormant stage of *P. vivax* is primaquine (PQ) [8]. Ethiopia adopted PQ in 2018. Currently, the national treatment guideline of Ethiopia includes three days of chloroquine (CQ) (total dose of 25 mg/kg base) plus 14 days of PQ (0.25 mg/kg/day, from day 0 to 13) for the treatment of uncomplicated *P. vivax* [9]. PQ administration is linked with varying degrees of hemolysis in patients with glucose 6-phosphate dehydrogenase deficiency (G6PDd). The prevalence of G6PDd is very low in Ethiopia [10], and PQ is rolled out without the need for G6PDd testing.

Chloroquine has been in use for several decades in Ethiopia. A recent report highlighted high rates of recurrent vivax parasitaemia on day 42 in CQ arm compared to CQ plus PQ arm [11]. This could be a challenge for the ambitious elimination efforts. In these studies, one of the challenges is there are no reliable tools to distinguish the source of recurrent vivax parasitaemia (relapse, recrudescence, or new infection) [12]. Following the first report of signs of resistance of *P. vivax* to CQ in 1989 [13] subsequent reports highlighted varying response [14–17]. The first CQ resistant *P. vivax* was reported from the highlands of Ethiopia in 1996 [18] followed by continued reports from different parts of the country [19–22]. Treatment failure rates were consistently below 10% in these studies except in one study (22%) [1, 23]. Globally, there is no evidence for the presence of PQ resistance. Confounding factors make the assessment of resistance to PQ difficult [24, 25]. Therefore, regular therapeutic efficacy monitoring studies are crucial for informed programmatic decision-making.

The aim of this study was to assess (i) the efficacy of CQ plus 14 days low dose of PQ as radical cure treatment for uncomplicated *P. vivax* and (ii) the haematologic response, PQ toxicity, gametocytaemia, fever and parasite clearance in 42 days follow up.

## Methods

### Study area

**Fig. 1:**
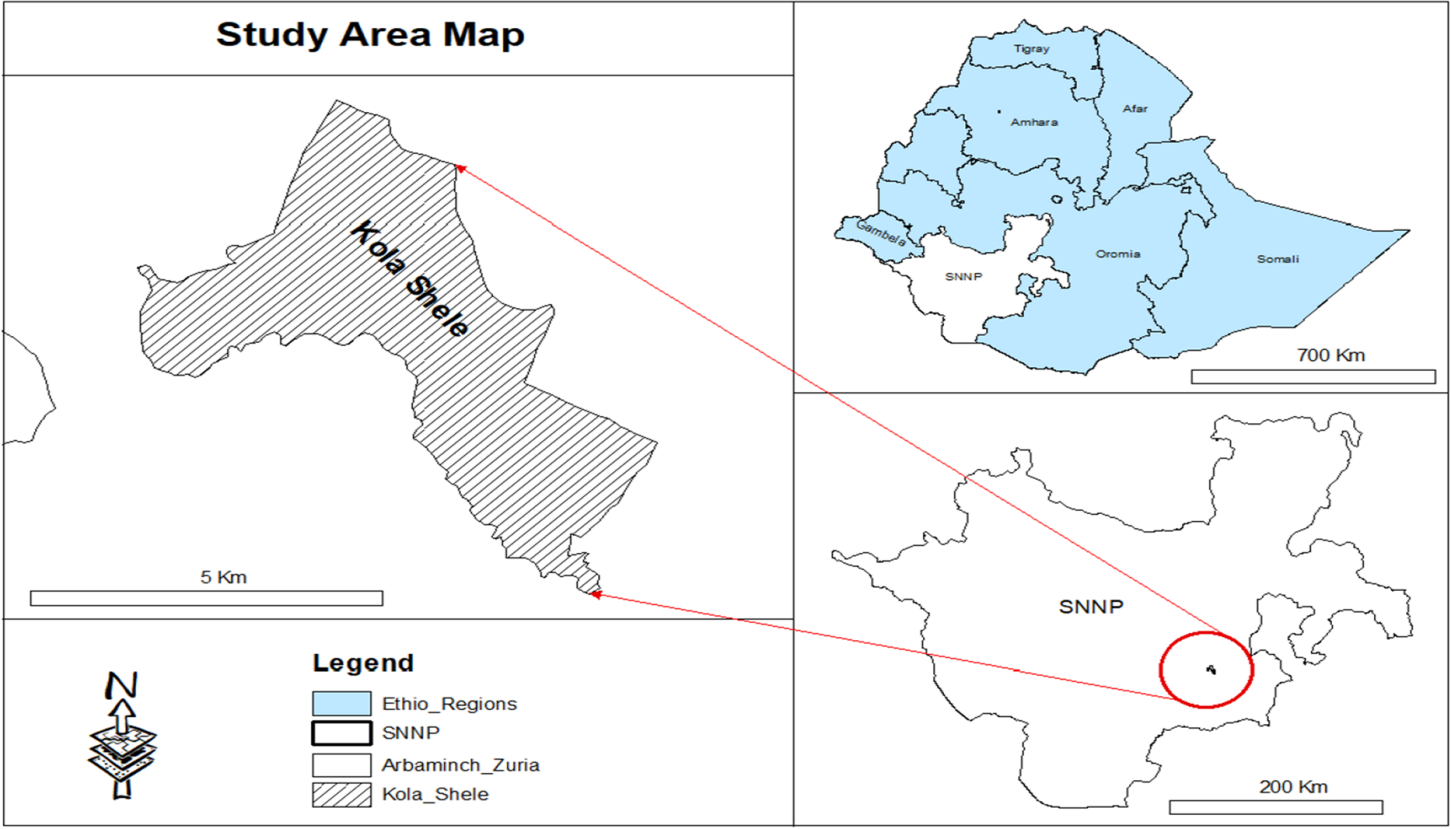
Map of the study area

A prospective longitudinal efficacy study was conducted between October 2019 and February 2020, at Kola Shele Health Centre, Arba Minch, Southern Ethiopia. *Anopheles arabiensis* is the major vector [26] in the area. Both *P. falciparum* and *P. vivax* are endemic with highly seasonal transmission (Fig. 1).

### Treatment and follow up

Treatment was given as per the national guideline [9]. Patients who vomited within 30 min were given the same dose of drugs and those who vomited twice were excluded. The first 3 days of treatments were directly observed and blister packs counted in subsequent visits: at days 7 and 14. Quality-assured drugs (Chloroquine phosphate, Medophgroup Batch no.9ME50 and Primaquine phosphate, Remedica Batch no.80643) obtained from the Ethiopian Pharmaceutical Supply Agency were used. Participants were advised to come to the health facility at any time during follow up if they showed signs and symptoms of malaria. Patients with recurrent parasitaemia of any *Plasmodium* species were treated according to the treatment guideline [9] and excluded from the study [27].

### Ethical clearance and patient recruitment

Ethical clearance was obtained from the Armauer Hansen Research Institute (AHRI/ALERT) (PO42/18) and the National Research Ethics Review Committee (MoSHE//RD 141/1097/19). Enrolment was made after explaining the purpose of the study and obtaining written informed consent. Consenting febrile patients (axillary temperature ≥ 37.5 or history of fever within the last 48 h) attending the outpatient department were screened as per the inclusion and exclusion criteria. Volunteer patients who fulfilled the following criteria were recruited: confirmed *P. vivax* mono infection (microscopically and CareStart™ Malaria RDT, Access Bio Inc), haemoglobin (Hb) level ≥ 5 g/dL, asexual parasitaemia ≥ 250 parasites/µL, aged above 6 months, ability to swallow oral medication, and a negative pregnancy test or not breastfeeding [27].

### Clinical procedure

A general physical examination was done by study physicians during enrolment. Baseline data, including demography, axillary temperature and anthropometric measures were recorded. At each visit, a symptom questionnaire and adverse or serious adverse events were recorded. At recruitment and during the scheduled follow up days, finger prick blood samples (300 µL) were collected using EDTA-microtainer tube (Becton Dickinson) for malaria diagnosis using RDT and thick and thin blood films, to prepare dried blood spots (DBS) on Whatman™ 3MM (VWR^®^) filter paper and measure haemoglobin (Hb) (days 0, 3, 14, 28, and 42) using portable spectrophotometer (Hemocue Hb 301+, Anglom, Sweden). Hillmen urine test was performed on days 1, 2, 3, 7, and 14 as per the national guideline [9] with slight modification. All female study participants aged 12 years and above were tested for pregnancy and pregnant women were excluded from the study.

### Laboratory procedures

Blood slides were stained with 10% Giemsa [27] for 10 min and read by two independent microscopists without knowledge of the RDT result. The number of asexual parasites was counted per 200 white blood cells (WBC) and parasitaemia estimated assuming a WBC count of 8000/µL. Microscopic gametocytes detection was also performed [27, 28]. The final parasite density was determined by taking the average of the parasitaemia of the two closest readings. Any discordant readings were re-examined by a third independent expert microscopists at Adama malaria training centre. Genomic DNA was extracted from DBS as described before using the Chelex-Saponin method [29]. Malaria species identification were done on day 0 and day of recurrent samples using nested polymerase chain reaction (nPCR) targeting the 18 S gene [30]. *Plasmodium vivax merozoite protein 3*α (*Pvmsp3α*) genotyping was done on paired samples from day 0 and day of recurrence using nPCR-restriction fragment length polymorphism (RFLP) method [31].

### Primary outcome

The primary endpoint was cumulative risk of *P. vivax* recurrence at day 42. Parasitological recurrence was classified as treatment failure on the day it occurred; whereas lost to follow up (LFU), withdrawals (WTH), and parasitaemia with a different species were censored on the last day of follow up. Treatment failures were categorized as (i) early treatment failure (ETF) (danger sign or severe malaria on days 1, 2 or 3 in the presence of parasitaemia; parasitaemia on day 2 higher than day 0, irrespective of axillary temperature; parasitaemia on day 3 with axillary temperature ≥ 37 ºC and parasitaemia on day 3 ≥ 25% of count on day 0); (ii) late clinical failure (LCF) (danger sign or sever malaria in the presence of parasitaemia on any day between day 4 and 28 or day 42 in patients who did not previously meet any of the criteria of early treatment failure and presence of parasitaemia in between days 4 and 28 or day 42 with axillary temperature ≥ 37 ºC in patients who did not previously meet any of the criteria of early treatment failure) and (iii) late parasitological failure (LPF) (presence of parasitaemia on any day between day 7 and 28 or day 42 with axillary temperature ≤ 37 ºC in patients who did not previously meet any of the criteria of early treatment failure or late treatment failure) and (iv) adequate clinical and parasitological response (ACPR) (absence of parasitaemia on day 28 or day 42, irrespective of axillary temperature in patients who did not previously meet any of the criteria of early treatment failure or late clinical failure or late parasitological failure according to the definition of WHO protocol [27]. All recurrences that occur in between day 28 to 42 were considered as relapses because of the intermediate endemicity of *P. vivax* malaria in the study area and the limitations in accurately distinguishing reinfection from actual relapse molecularly [12].

Other secondary endpoints were defined as Hb recovery (days 0, 3, 14, 28, and 42) and drug adverse events (on days 1, 2, 3, 7 and 14) fever and asexual parasite clearance (on days 1, 2 and 3) and gametocytaemia at follow up days 1, 2 and 3.

### Statistical analysis

Data were double entered using Microsoft Excel sheet and analyzed using the WHO Microsoft Excel and STATA v.14. Frequency counts with percentages (for gender, gametocyte detection, use of malaria infection prevention, PCR genotyping, hillmen urine test and adverse events), median with ranges (for age and haemoglobin level), arithmetic mean with standard deviations (for temperature and haemoglobin level) and geometric mean with ranges (for asexual parasite) were done. Change in mean haemoglobin level was compared using Wilcoxon signed-rank test. The cumulative incidence of failure on days 28 and 42 was assessed by survival probability analysis using Kaplan-Meier method. Anyone lost to follow up or withdrew or presenting with *P. falciparum* infection was censored.

## Results

### Treatment outcome

**Fig. 2:**
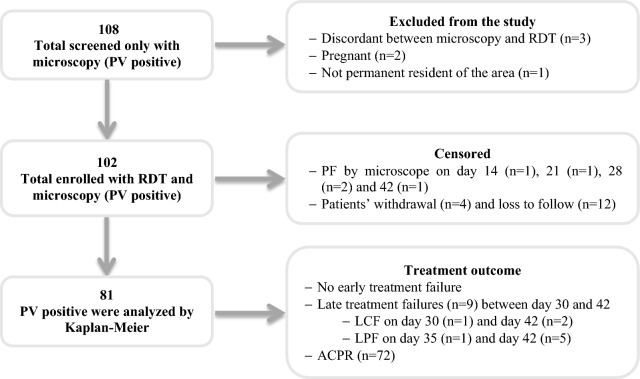
Flow chart of patient allocation for 42 days of follow up. Of the 108 microscopy *P. vivax* mono-infections, 3 were *P. falciparum* by RDT (discordant), 2 were pregnant and 1 volunteer was not resident at the study area and thus excluded from the study. During follow up, five turned *P. falciparum* positive on days 14 (n = 1), 21 (n = 1), 28 (n = 2) and 42 (n = 1). Sixteen cases were lost to follow up or withdrawn from the study and thus excluded from the Kaplan-Meier analysis. Of the 81 remaining patients; 9 had treatment failure on day 30 (n = 1), 35 (n = 1) and 42 (n = 7). *PF* *P. falciparum,* *PV* *P. vivax,* *RDT* rapid diagnostic test, *ACPR* adequate clinical and parasitological response, *LCF* late clinical failure, *LPF* late parasitological failure

Recurrence of *P. vivax* parasitaemia was not detected until day 28 of the follow up. There was no early treatment failure. Seventy-two (88.9%) patients had adequate clinical and parasitological response (ACPR) on day 42. The cumulative incidence of failure at day 42 was 10.9% (95% CI 5.8–19.9%). Sixteen patients were lost to follow up (n = 12) and withdrew consent/assent (n = 4) (Fig. 2).

### Genotyping of ***P. vivax*** parasites at recruitment (day 0) and day of recurrence

**Fig. 3:**
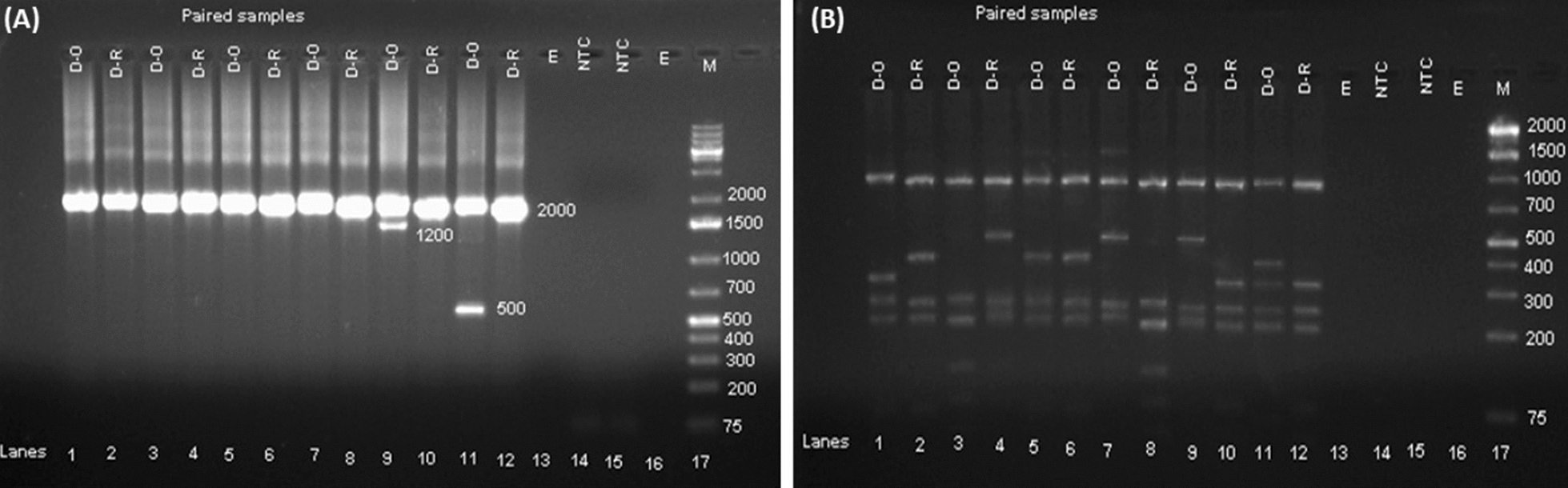
PCR products and restriction fragment length polymorphism patterns of *Pvmsp3α* gene. The PCR product of *Pvmsp3α* gene is shown for 12 *P. vivax* paired samples (**A**) together with digested products with *Hha* I enzyme (**B**). *D-0* Day 0, *D-R* Day recurrent, *NTC* negative template control, *E*  empty and *M*  1-Kb plus DNA marker

Among the 102 microscopically and RDT *P. vivax* positive patients on day of enrollment, 93 (91.2%) were confirmed to be *P. vivax* with the rest being *P. falciparum* (2, 1.9%), mixed species (6, 5.9%), or negative (1, 1.0%) using 18 S based nPCR. Fourteen patients became microscopy positive for *Plasmodium* parasites within the 42 days of follow up (5 *P. falciparum* and 9 *P. vivax* infected). Of the 5 *P. falciparum* microscopy positive patients, one was negative by nPCR whilst all *P. vivax* infections were confirmed. Paired samples from day 0 and day of recurrent parasitaemia of the 3 LCF and 6 LPF were genotyped targeting *Pvmsp3α* gene. Based on the length variants of the PCR products [31], out of the 9 paired (n = 18) samples, three allele sizes were detected: 88.9% (16/18) were type A (1900 bp), 5.5% (1/18) was type B (1500 bp) and 5.5% (1/18) was type D (500 bp). Type B and the rare Type D were detected as mixed infection with variant Type A (Fig. 3a) whereas all the remaining day 0 and recurrent samples were only type A. Fragments between 85 − 1000 bp were observed after PCR product digestion with the *Hha* I enzyme. Overall, eleven different restriction patterns were found. Identical restriction digestion band patterns on day 0 and day of recurrence were observed only for two samples (Fig. 3b). The restriction digestion band patterns for the remaining 7 paired samples were different between day 0 and day of recurrence (Fig. 3b).

The mean axillary temperature and Hb concentration at baseline were 37.4 °C and 11.8 g/dL, respectively. The highest parasite count was observed for children under 15 years old. On the day of recruitment, 44.1% (45/102) of the participants had microscopically detectable gametocytes. The vast majority of participants reported that they did not have/use malaria infection prevention tools (Table 1).

**Table 1:** Demographic and clinical characteristics of *P. vivax* patients at the day of enrollment

Patient characteristics			Values	
Male gender, n (%)			51 (50.0%)	
Age (years), median (range)			13.5 (5–44)	
Axillary temperature, mean (SD)			37.4 ℃ (1.3)	
Haemoglobin (g/dL), mean (SD)			11.8 (2.2)	

Microscopy asexual parasite density, geometric mean (range) in age groups				
5 to 15 year (n = 54)			8061.5/µL (246-32233)	
> 15 year (n = 48)			4427/µL (541-31000)	
Patients presenting with gametocytes			44.1% (45/102)	

Use of malaria infection prevention, n (%)				
Household with a bed net	2 (2.0%)			
Patient who slept under bed net on the night before recruitment	1 (1.0%)			
Households sprayed with insecticides	35 (34.7%)			

*SD* standard deviation, *n* numbers, *g/dL* gram per deciliter

### Fever, parasite and gametocyte clearance

Treatment with CQ plus PQ resulted in fast resolution of fever and clearance of parasites and gametocytes. Out of the 49 febrile cases, on day of enrolment, only 8.3% (4/48) were febrile on day one. Fever subsided in all patients on day 2 but on day 3 one patient was found febrile. Asexual parasites were detected in half of the patients on day 1 (53.46%, 54/101) and only in three patients (3.13%) on day 2. Parasites were cleared completely in all patients on day 3. Similarly, gametocyte clearance mirrored asexual parasite clearance as detected by microscopy. Gametocytes were detected only in 9.90% (10/101) patients on day 1 and only in one patient on day 2. After day 3, no gametocytes were detected (Table 2).

**Table 2:** Fever, parasite and gametocyte clearance

Time point	Day 0		Day 1		Day 2		Day 3	
Age group (Years)	5–15	> 15	5–15	> 15	5–15	> 5	5–15	> 15
Cases with fever 37.5 ℃, n (%)	27/54 (26.47%)	22/48 (21.57%)	2/26 (4.17%)	2/22 (4.17%)	0/26 (0.00%)	0/21 (0.00%)	0/26 (0.00%)	1/21 (2.13%)
Cases with parasitaemia, n (%)*	54/54 (100.00%)	48/48 (100.00%)	28/53 (27.72%)	26/48 (25.74%)	3/50 (3.13%)	0/46 (0.00%)	0/50 (0.00%)	0/44 (0.00%)
Cases with gametocytes, n (%)*	23/54 (22.55%)	22/48 (21.57%)	8/45 (7.92%)	2/46 (1.98%)	1/50 (1.04%)	0/46 (0.00%)	0/50 (0.00%)	0/44 (0.00%)

*Detected by microscopy

### Haemoglobin (hb) and Hillmen urine test measurement

A statistically significant difference (*P* < 0.001) was observed in the Hb level between day 0 and 42; the median Hb concentration on day 42 (13.40 g/dL, range 8.40–18.00 g/dL) was higher than baseline (11.85 g/dL, range 5.20–16.70 g/dL) (Table 3). The Hillmen test rank were under the score of 5 on days 1, 2, 3, 7 and 14 (Table 4).

**Table 3:** Haemoglobin recovery

Follow up days	Median (min-max) (g/dL)	*P value*
Day 0	11.85 (5.20–16.70)	< 0.001
Day 3	11.85 (7.60–16.60)	
Day 14	12.70 (9.70–16.20)	
Day 28	13.10 (9.70–17.40)	
Day 42	13.40 (8.40–18.00)

**Table 4:** Hillmen Urine Test result

Rank	Follow up days				
	Day 1, n (%)	Day 2, n (%)	Day 3, n (%)	Day 7, n (%)	Day 14, n (%)
1	43 (42.57)	59 (61.46)	73 (77.66)	85 (91.40)	82 (91.11)
2	17 (16.83)	18 (18.75)	19 (20.21)	8 (8.60)	8 (8.89)
3	26 (25.74)	14 (14.58)	2 (2.13)	0 (0.00)	0 (0.00)
4	15 (14.85)	5 (5.21)	0 (0.00)	0 (0.00)	0 (0.00)

### Adverse events

The most frequent adverse events on day 1 were complaints of fever (11.88%), headache (8.91%), nausea (5.69%), abdominal pain (4.95%), chill (3.96%) and vomiting (2.97%). The incidence of adverse events declined from day 1 onwards. Dark-colored urine was not reported except on day 1 in two (1.98%) patients (Table 5).

**Table 5:** Adverse events recorded

Adverse events	Follow up days				
	Day 1	Day 2	Day 3	Day 7	Day 14
Fever	12 (11.88%)	0 (0.00%)	1 (1.05%)	1 (1.08%)	2 (2.20%)
Chill	4 (3.96%)	0 (0.00%)	0 (0.00%)	0 (0.00%)	0 (0.00%)
Headache	9 (8.91%)	0 (0.00%)	1 (1.05%)	0 (0.00%)	3 (3.30%)
Nausea	6 (5.94%)	1 (1.03%)	4 (4.21%)	0 (0.00%)	1 (1.10%)
Vomiting	3 (2.97%)	1 (1.03%)	1 (1.05%)	0 (0.00%)	0 (0.00%)
Abdominal pain	5 (4.95%)	3 (3.09%)	2 (2.11%)	2 (2.15%)	1 (1.10%)
Diarrhoea	0 (0.00%)	0 (0.00%)	0 (0.00%)	0 (0.00%)	0 (0.00%)
Cough	1 (0.99%)	0 (0.00%)	0 (0.00%)	0 (0.00%)	0 (0.00%)
Weakness	2 (1.98%)	2 (2.06%)	0 (0.00%)	0 (0.00%)	0 (0.00%)
Body ache	0 (0.00%)	0 (0.00%)	0 (0.00%)	0 (0.00%)	0 (0.00%)
Itching	0 (0.00%)	2 (2.06%)	0 (0.00%)	0 (0.00%)	0 (0.00%)
Rash	0 (0.00%)	0 (0.00%)	0 (0.00%)	0 (0.00%)	0 (0.00%)
Eye discharge	0 (0.00%)	0 (0.00%)	0 (0.00%)	0 (0.00%)	0 (0.00%)
Mouth lesion	0 (0.00%)	0 (0.00%)	1 (1.05%)	0 (0.00%)	0 (0.00%)
Dark urine	2 (1.98%)	0 (0.00%)	0 (0.00%)	0 (0.00%)	0 (0.00%)

## Discussion

Following the rollout of PQ for radical cure of *P. vivax* in Ethiopia in 2018 to support its ambitious elimination efforts, this study demonstrated that administration of PQ is safe and efficacious. Of the 81 patients that completed the follow up early treatment failure was not observed. Infection was detected only after day 28. One hundred two patients positive for *P. vivax* by microscopy and RDT were followed for 42 days post treatment with CQ and a 14 days low dose of PQ in a treatment efficacy study (TES) following the WHO protocol in a *P. falciparum* and *P. vivax* co-endemic setting in Ethiopia [32]. None of the recurrent *P. vivax* infections occurred within 28 days of follow up [32] implying that in this study there were no CQ treatment failure. A recent study in the same study area reported 3.80% treatment failure following a CQ alone treatment study in a 28 days follow up [22]. In present study at the same study area the addition of low dose PQ for 14 days to the standard CQ regimen have minimized the recurrence of parasitaemia before day 28 due to the asexual blood stage activity of the PQ [33]. Studies from other parts of Ethiopia reported reduction of the recurrence of parasitaemia within 28 days following CQ plus PQ administration [11, 20]. Similar study indicated in Brazil that using similar regimens with ACPR of 100% at day 28 [34]. Similarly, meta-analysis by Commons et al. [35] indicated that the addition of PQ to CQ reduces early recurrences before day 42 by 90% compared with CQ alone. Though in this study, the recurrences of parasitaemia was documented from day 30 onwards. The longer the follow up is the more likely the chance is to detect recurrent infections [20].

True PQ resistance is really difficult to build, there are case reports of recurring vivax malaria despite an adequate dose of PQ administered with an effective blood schizontocidal agent [36]. It has been suggested that recurrence of parasitaemia in the three to six months after PQ is likely to be a relapse [37, 38]. In tropical regions endemic for *P. vivax*, including Ethiopia, the risk of *P. vivax* relapse is generally high [39] and it is one of the major challenges in controlling and eliminating vivax malaria [40]. In the present study, the recurrent parasitaemia that occurred between day 30 and 42 could be a result of relapse because it is rare to be re-infected with the same clone. Though, studies confirmed that therapeutic levels CQ could persist in blood until days 21 to 35 after the start of treatment [41, 42] and in the present study the stipulated study time was 42 days and patients were treated directly observed with the standard doses of CQ and expect that lingering CQ in the blood to supress the the relapsed vivax malaria. The pitfall in this TES is CQ blood level wasn’t measured to investigate the presence of CQ resistant vivax malaria in the recurrent samples.

PQ failure and *P. vivax* relapse is a major global public health concern [25, 43]. Resistance or tolerance to PQ by *P. vivax* has been documented in Somalia and South East Asia [43–45]. True treatment failure with PQ is difficult to define due to the presence of confounding factors [24, 25]. Potential risk factors for the occurrences of relapse after PQ treatment could be age and baseline anti-malarial immunity and quality of PQ drug being used [25], dose of treatment [46, 47], duration of therapy [48], individual variations in CYP2D6 [49, 50], adherence to treatment [24, 25] and re-infection [51]. Though there are no data on the level of anti-malarial immunity in the study area, in the present study relapse has occurred in 7 patients under 15 years old and in two patients who are above 15 years old indicating that in young patients their immunity might be weak to suppress relapse when compared with adults. The quality of the drugs was ruled out (since the drugs were obtained from government which is considered to be a standard one) and there were no report of counterfeit anti-malarial drugs. All cases were treated according to the national guideline [9]. PQ is a pro-drug that is required to be metabolized by liver for the generation of molecules displaying activity against hypnozoites [52]. Though, it was reported that in Ethiopia about 29% of the population is known to carry active CYP2D6 gene duplications and multiplications associated with increased enzyme activity [53], and there is no report on the effect of CYP2D6 activity for PQ metabolism in Ethiopia. Though there are other factors impact the risk of relapse after PQ therapy, it was suggested that almost all reports of malaria resistant to PQ are associated with lack of such supervision [43]. Regarding adherence to treatment, in this s study for the first four days (day 0 to day 3) treatment with CQ and PQ were directly observed and from day 4 up to 13 PQ was given to the patients to be taken at home and adherence was assessed by tablet review at the subsequent visit. Therefore, for PQ treatment from days 4 to 13, absolute confidence with regard to adherence could not be maintained. However, based on the information obtained and tablet review at subsequent visit from patients, lack of adherence is a less probable reason that resulted PQ treatment failure/relapse.

Nine samples (8.8%) from day 0 were discordant with PCR: six of them were mixed; two were *P. falciparum* and one negative. During follow up from among these discordant nPCR mixed samples, one case had *P. falciparum* infection on day 28 and two were LFU on day 2 and 7. For the rest of cases whether they were mixed, *P. falciparum* or negative for PCR, parasitaemia were not detected until the end of the study. This achievement might be attributable to the schizonticidal effect of PQ on both *P. vivax* and *P. falciparum* [54]. According to the national treatment guideline [9], these discordant malaria cases would have been considered as misdiagnosed and mistreated. During follow up 14 parasitaemia cases were detected. Nine were recurrent *P. vivax* cases and five were *P. falciparum* infection: *P. falciparum* infections were detected after day 14 which were *P. vivax* during recruitment. In most malaria-endemic regions, the gold standard for diagnosing malaria is still microscopic examination of stained blood smears. However, in regions where *P. falciparum* and *P. vivax* are co-endemic, widespread misdiagnosis has been documented [55–57]. Misdiagnosis and mistreatment of malaria cases would be one of the major challenges for the elimination goal set by the country.

PCR genotyping of *P. vivax* is ambiguous to assign the causes of recurrence to reinfection, recrudescence or relapse [12, 58]. In this study, *Pvmsp3α* genotyping result indicated that among the 9 paired samples (day 0 and day of recurrent), 7 of them had Type A alleles and 2 samples had mixed infections with Type A and Type B alleles for the first sample and Type A and D alleles for the second sample on day 0 but on day of recurrence only Type A allele was detected for the two samples. *Pvmsp3α* genotyping results were used to calculate adjusted cumulative risk of recurrence by day 42 by censoring for heterologous infections [59]. In this study, after PCR adjustment, treatment failure were assigned to those isolates with identical genotypes [60]. *Pvmsp3α* genotyping for the two paired samples that were Type A size variant, the restriction digestion bands were identical on day 0 and day of recurrence. These identical clones were detected on days 30 and 42 and the rest of recurrent *P. vivax* parasites were heterologous. As reinfection with a similar genotype had a probability ≤ 0.002 [61], recurrences of the same genotype were considered all to be relapses. It was assumed that genetically heterologous early relapses shared similar periodicity to the genetically homologous relapses in relation to the primary infection [58, 61, 62]. This phenomenon suggests that these patients, who responded suitably to the treatment, had relapse since similar patterns or shared bands were found in the paired samples. On the other hand, these patients stayed in the same endemic zone all the time, making it possible for them to acquire a new infection. However, these results must be interpreted with caution since they could also have presented a relapse caused by activation of heterologous hypnozoites that persisted in the liver from the first infection. Based on the interpretation of nPCR-RFLP, those that were considered as treatment failures/relapse before PCR adjustments could be classified as new infections due to the difference in restriction digestion bands between day 0 and recurrent samples. PCR adjusted treatment failure was 2.40% which is lower than PCR unadjusted treatment failure (10.90%); possibly PCR adjustment could lead to overestimation of therapeutic efficacy of PQ and hence care should be taken while interpretation by considering the different confounding factors that could lead to recurrence of parasitaemia and also dosing difference could be the causes of *P*. *vivax* relapse after PQ therapy [7] low dose of PQ was used as per the treatment guideline without the need for G6PDd testing [9] and perhaps in the near future if G6PDd testing is included in the treatment guideline to reduce the risk of hemolysis by PQ and by increasing the dose of PQ the probability of detecting *P*. *vivax* relapse after PQ therapy could be reduced. The limitation in present study is that there is no information on the genetic diversity of *P*. *vivax* circulating in the study area to increase the level of confidence in judging reinfection with the same or different clone.

The present study showed that the combined CQ plus PQ treatment is well tolerated and resulted in early clearance of both the asexual and sexual stages.

## Conclusions

Co-administration of CQ with PQ in the study area is well tolerated and there were no recurrence of *P. vivax* before 28 days of follow up. Interpretation of CQ plus PQ efficacy should be done with caution especially when the recurrent parasitaemia occurs after day 28. Given the observed failures in later days with the combination regimen, it would be important to conduct therapeutic efficacy studies with appropriate design to rule out CQ or PQ drug resistance in the study area.

## Data Availability

Data can be accessed from the corresponding author through email.

## References

[1] WHO (2021). World malaria report 2021.

[2] PMI MOP. President’s Malaria Initiative Malaria operational plan. Addis Ababa, Ethiopia; 2018.

[3] Woyessa A, Deressa W, Ali A, Lindtjørn B (2012). Prevalence of malaria infection in Butajira area, south-central Ethiopia. Malar J.

[4] Keffale M, Shumie G, Behaksra SW, Chali W, Hoogen LLvd, Hailemeskel E (2019). Serological evidence for a decline in malaria transmission following major scale-up of control efforts in a setting selected for Plasmodium vivax and Plasmodium falciparum malaria elimination in Babile district, Oromia, Ethiopia. Trans R Soc Trop Med Hyg.

[5] Howes RE, Battle KE, Mendis KN, Smith DL, Cibulskis RE, Baird JK (2016). Global epidemiology of *Plasmodium vivax*. Am J Trop Med Hyg.

[6] Nadjm B, Behrens RH (2012). Malaria: an update for physicians. Infect Dis Clin North Am.

[7] Baird JK (2004). Chloroquine resistance in *Plasmodium vivax*. Antimicrob Agents Chemother.

[8] WHO (2015). Guidelines for the treatment of malaria.

[9] Federal Ministry of Health. National malaria guidelines. In: Malaria diagnosis and treatment. 4th ed. Addis Ababa, Ethiopia. 2018.

[10] Assefa A, Ali A, Deressa W, Tsegaye W, Abebe G, Sime H (2018). Glucose-6-phosphate dehydrogenase (G6PD) deficiency in Ethiopia: absence of common african and Mediterranean allelic variants in a nationwide study. Malar J.

[11] Abreha T, Hwang J, Thriemer K, Tadesse Y, Girma S, Melaku Z (2017). Comparison of artemether-lumefantrine and chloroquine with and without primaquine for the treatment of *Plasmodium vivax* infection in Ethiopia: a randomized controlled trial. PLoS Med.

[12] Chen N, Auliff A, Rieckmann K, Cheng Q (2007). Relapses of *Plasmodium vivax* infection result from clonal hypnozoites activated at predetermined intervals. J Infect Dis.

[13] Rieckmann K, Davis D, Hutton D (1989). *Plasmodium vivax* resistance to chloroquine?. Lancet.

[14] Baird JK, Basri H, Bangs MJ, Subianto B, Patchen LC, Hoffman SL (1991). Resistance to chloroquine by *Plasmodium vivax* in Irian Jaya, Indonesia. Am J Trop Med Hyg.

[15] Myat-Phone-Kyaw, Myint-Oo, Myint-Lwin, Thaw-Zin, Kyin-Hla-Aye, Nwe-Nwe-Yin. Emergence of chloroquine-resistant *Plasmodium vivax* in Myanmar (Burma). Trans R Soc Trop Med Hyg. 1993;87:687. 10.1016/0035-9203(93)90294-z 8296378

[16] Garg M, Gopinathan N, Bodhe P, Kshirsagar N (1995). Vivax malaria resistant to chloroquine: case reports from Bombay. Trans R Soc Trop Med Hyg.

[17] Phillips EJ, Keystone JS, Kain KC (1996). Failure of combined chloroquine and high-dose primaquine therapy for *Plasmodium vivax* malaria acquired in Guyana, South America. Clin Infect Dis.

[18] Tulu AN, Webber RH, Schellenberg JA, Bradley DJ (1996). Failure of chloroquine treatment for malaria in the highlands of Ethiopia. Trans R Soc Trop Med Hyg.

[19] Teka H, Petros B, Yamuah L, Tesfaye G, Elhassan I, Muchohi S (2008). Chloroquine-resistant *Plasmodium vivax* malaria in Debre Zeit, Ethiopia. Malar J.

[20] Yeshiwondim AK, Tekle AH, Dengela DO, Yohannes AM, Teklehaimanot A (2010). Therapeutic efficacy of chloroquine and chloroquine plus primaquine for the treatment of *Plasmodium vivax* in Ethiopia. Acta Trop.

[21] Ketema T, Getahun K, Bacha K (2011). Therapeutic efficacy of chloroquine for treatment of *Plasmodium vivax* malaria cases in Halaba district, South Ethiopia. Parasit Vectors.

[22] Getachew S, Thriemer K, Auburn S, Abera A, Gadisa E, Aseffa A (2015). Chloroquine efficacy for *Plasmodium vivax* malaria treatment in southern Ethiopia. Malar J.

[23] WHO (2020). World malaria report 2020: 20 years of global progress and challenges.

[24] Ashley EA, Recht J, White NJ (2014). Primaquine: the risks and the benefits. Malar J.

[25] Rishikesh K, Saravu K (2016). Primaquine treatment and relapse in *Plasmodium vivax* malaria. Pathog Glob Health.

[26] Mohammed H, Mindaye T, Belayneh M, Kassa M, Assefa A, Tadesse M (2015). Genetic diversity of *Plasmodium falciparum* isolates based on MSP-1 and MSP-2 genes from Kolla-Shele area, Arbaminch Zuria District, southwest Ethiopia. Malar J.

[27] WHO (2009). Methods for surveillance of antimalarial drug efficacy.

[28] WHO (2010). Basic malaria microscopy: tutor’s guide.

[29] Baidjoe A, Stone W, Ploemen I, Shagari S, Grignard L, Osoti V (2013). Combined DNA extraction and antibody elution from filter papers for the assessment of malaria transmission intensity in epidemiological studies. Malar J.

[30] Snounou G, Viriyakosol S, Jarra W, Thaithong S, Brown KN (1993). Identification of the four human malaria parasite species in field samples by the polymerase chain reaction and detection of a high prevalence of mixed infections. Mol Biochem Parasitol.

[31] Bruce MC, Galinski MR, Barnwell JW, Snounou G, Day KP (1999). Polymorphism at the merozoite surface protein-3alpha locus of *Plasmodium vivax*: global and local diversity. Am J Trop Med Hyg.

[32] WHO (2002). Monitoring antimalarial drug resistance: report of a WHO consultation, Geneva, Switzerland, 3–5 December 2001.

[33] Pukrittayakamee S, Vanijanonta S, Chantra A, Clemens R, White NJ (1994). Blood stage antimalarial efficacy of primaquine in *Plasmodium vivax* malaria. J Infect Dis.

[34] Negreiros S, Farias S, Viana GMR, Okoth SA, Chenet SM, de Souza TMH (2016). Efficacy of chloroquine and primaquine for the treatment of uncomplicated *Plasmodium vivax* malaria in Cruzeiro do sul, Brazil. Am J Trop Med Hyg.

[35] Commons RJ, Simpson JA, Thriemer K, Humphreys GS, Abreha T, Alemu SG (2018). The effect of chloroquine dose and primaquine on *Plasmodium vivax r*ecurrence: a WorldWide Antimalarial Resistance Network systematic review and individual patient pooled meta-analysis. Lancet Infect Dis.

[36] Reddy P, Flaherty JP (2006). *Plasmodium vivax* malaria relapses after primaquine prophylaxis. Emerg Infect Dis.

[37] Alves FP, Durlacher RR, Menezes MJ, Krieger H, Silva L, Camargo EP (2002). High prevalence of asymptomatic *Plasmodium vivax* and *Plasmodium falciparum* infections in native amazonian populations. Am J Trop Med Hyg.

[38] Carmona-Fonseca J, Álvarez G, Blair S (2006). *Plasmodium vivax* malaria: treatment of primary attacks with primaquine, in three different doses, and a fixed dose of chloroquine, Antioquia, Colombia, 2003–2004. Biomédica.

[39] Karunajeewa HA, Mueller I, Senn M, Lin E, Law I, Gomorrai PS (2008). A trial of combination antimalarial therapies in children from Papua New Guinea. N Engl J Med.

[40] White NJ, Imwong M (2012). Relapse. Adv Parasitol.

[41] Coatney GR, Ruhe DS, Cooper WC, Josephson ES, Young MD (1949). Studies in human malaria. X. The protective and therapeutic action of chloroquine (SN 7618) against St. Elizabeth strain vivax malaria. Am J Hyg.

[42] Lee SJ, McGready R, Fernandez C, Stepniewska K, Paul MK, Viladpai-Nguen SJ (2008). Chloroquine pharmacokinetics in pregnant and nonpregnant women with vivax malaria. Eur J Clin Pharmacol.

[43] Baird JK, Hoffman SL (2004). Primaquine therapy for malaria. Clin Infect Dis.

[44] Smoak BL, DeFraites RF, Magill AJ, Kain KC, Wellde BT (1997). *Plasmodium vivax* infections in US Army troops: failure of primaquine to prevent relapse in studies from Somalia. Am J Trop Med Hyg.

[45] Wilairatana P, Silachamroon U, Krudsood S, Singhasivanon P, Treeprasertsuk S, Bussaratid V (1999). Efficacy of primaquine regimens for primaquine-resistant *Plasmodium vivax* malaria in Thailand. Am J Trop Med Hyg.

[46] Baird JK (2009). Resistance to therapies for infection by *Plasmodium vivax*. Clin Microbiol Rev.

[47] Pukrittayakamee S, Imwong M, Chotivanich K, Singhasivanon P, Day NPJ, White NJ (2010). A comparison of two short-course primaquine regimens for the treatment and radical cure of *Plasmodium vivax* malaria in Thailand. Am J Trop Med Hyg.

[48] Fernando D, Rodrigo C, Rajapakse S (2011). Primaquine in vivax malaria: an update and review on management issues. Malar J.

[49] Bennett JW, Pybus BS, Yadava A, Tosh D (2013). Primaquine failure and cytochrome P-450 2D6 in Plasmodium vivax malaria. N Engl J Med.

[50] Marcsisin SR, Reichard G, Pybus BS (2016). Primaquine pharmacology in the context of CYP 2D6 pharmacogenomics: current state of the art. Pharmacol Ther.

[51] Goller JL, Jolley D, Ringwald P, Biggs B-A (2007). Regional differences in the response of *Plasmodium vivax* malaria to primaquine as anti-relapse therapy. Am J Trop Med Hyg.

[52] Popovici J, Tebben K, Witkowski B, Serre D (2021). Primaquine for *Plasmodium vivax* radical cure: what we do not know and why it matters. Int J Parasitol Drugs Drug Resist.

[53] Aklillu E, Persson I, Bertilsson L, Johansson I, Rodrigues F, Ingelman-Sundberg M (1996). Frequent distribution of ultrarapid metabolizers of debrisoquine in an ethiopian population carrying duplicated and multiduplicated functional CYP2D6 alleles. J Pharmacol Exp Ther.

[54] Schmidt L (1969). Chemotherapy of the drug-resistant malarias. Annu Rev Microbiol.

[55] Barber BE, William T, Grigg MJ, Yeo TW, Anstey NM (2013). Limitations of microscopy to differentiate Plasmodium species in a region co-endemic for *Plasmodium falciparum*, *Plasmodium vivax* and *Plasmodium knowlesi*. Malar J.

[56] Coleman RE, Maneechai N, Rachaphaew N, Kumpitak C, Miller RS, Soyseng V (2002). Comparison of field and expert laboratory microscopy for active surveillance for asymptomatic *Plasmodium falciparum* and *Plasmodium vivax* in western Thailand. Am J Trop Med Hyg.

[57] McKenzie FE, Sirichaisinthop J, Miller RS, Gasser RA, Wongsrichanalai C (2003). Dependence of malaria detection and species diagnosis by microscopy on parasite density. Am J Trop Med Hyg.

[58] Imwong M, Snounou G, Pukrittayakamee S, Tanomsing N, Kim JR, Nandy A (2007). Relapses of *Plasmodium vivax* infection usually result from activation of heterologous hypnozoites. J Infect Dis.

[59] Price RN, Auburn S, Marfurt J, Cheng Q (2012). Phenotypic and genotypic characterisation of drug-resistant *Plasmodium vivax*. Trends Parasitol.

[60] Véron V, Legrand E, Yrinesi J, Volney B, Simon S, Carme B (2009). Genetic diversity of msp3α and msp1 _b5 markers of *Plasmodium vivax* in French Guiana. Malar J.

[61] Kim J-R, Nandy A, Maji AK, Addy M, Dondorp AM, Day NP (2012). Genotyping of *Plasmodium vivax* reveals both short and long latency relapse patterns in Kolkata. PLoS ONE.

[62] Imwong M, Boel ME, Pagornrat W, Pimanpanarak M, McGready R, Day NP (2012). The first *Plasmodium vivax* relapses of life are usually genetically homologous. J Infect Dis.

